# High productivity and multilayered circulation in the Late Cretaceous Arctic Ocean

**DOI:** 10.1126/sciadv.aec4895

**Published:** 2026-03-06

**Authors:** Shan Liu, David Van Rooij, Meiting Chen, Haiyu Fan, Guo Li, Yutong Shi, Yujie Liu, Zhi Lin Ng, Kunwen Luo, Li’e Lin, Haiteng Zhuo

**Affiliations:** ^1^ School of Marine Sciences, Sun Yat-sen University, 519082 Zhuhai, China.; ^2^ Guangdong Provincial Key Laboratory of Marine Resources and Coastal Engineering, 519082 Zhuhai, China.; ^3^ Department of Geology, Ghent University, Campus Sterre (building S8), Krijgslaan 281, B-9000 Gent, Belgium.; ^4^ State Key Laboratory of Marine Geology, Tongji University, 200092 Shanghai, China.

## Abstract

The Arctic Ocean plays a pivotal role in global climate, yet its circulation under greenhouse conditions remains poorly constrained. Seismic, sedimentological, and drilling evidence from the Chukchi Shelf reveals large contourite drifts and bathyal carbonate mounds dated to the Campanian to Maastrichtian [~80 to 66 million years ago (Ma)], indicating persistent bottom currents and high productivity. These features point to the presence of a regionally sourced Boreal deep-water mass, likely driven by seasonal sea-ice formation and brine rejection. Bathyal carbonate mound development was sustained by tidal mixing, localized upwelling, and well-oxygenated conditions following Oceanic Anoxic Event 3. This vigorous circulation system collapsed at the Cretaceous-to-Paleogene boundary, coinciding with closure of the Western Interior Seaway and reorganization of global circulation. Our findings challenge the prevailing view of a sluggish, surface-dominated Late Cretaceous Arctic, demonstrating instead that it supported deep-water formation and localized carbonate factories, highlighting its key role in high-latitude climate dynamics under greenhouse conditions.

## Teaser

Late Cretaceous Arctic sedimentary features reveal vigorous ocean circulation, overturning view of a sluggish greenhouse ocean.

## INTRODUCTION

The Late Cretaceous represents an extreme greenhouse interval in Earth’s climate history, characterized by high atmospheric CO_2_ concentrations, global warmth, and the absence of permanent polar ice caps (*1*). Oceanic circulation patterns during this period were fundamentally different from those of the modern ocean (*2*), yet they still played a crucial role in governing sedimentary processes and sustaining polar marine ecosystems under greenhouse conditions (*3*). High eustatic sea levels facilitated the expansion of extensive epicontinental seaways and marginal ocean basins, including the nearly semienclosed Arctic basin (*4*).

During the Late Cretaceous, the Arctic Ocean was enclosed by bordered continental landmasses and connected to adjacent seas only through narrow marine gateways, such as the Western Interior Seaway and the proto–North Atlantic straits (*5*, *6*). Previous interpretations have described the Late Cretaceous Arctic Ocean as a high temperature and warm, stratified basin, characterized by episodic sea-ice cover and restricted water mass exchange (*7*, *8*). However, existing evidence for bottom-current activity for this period is sparse and largely indirect. Limited accessibility and connectivity with surrounding oceans and the scarcity of paleocirculation reconstructions have hindered efforts to evaluate the Arctic’s role in global nutrient cycling, deep-water formation, and marine productivity during this period.

**Fig. 1.**
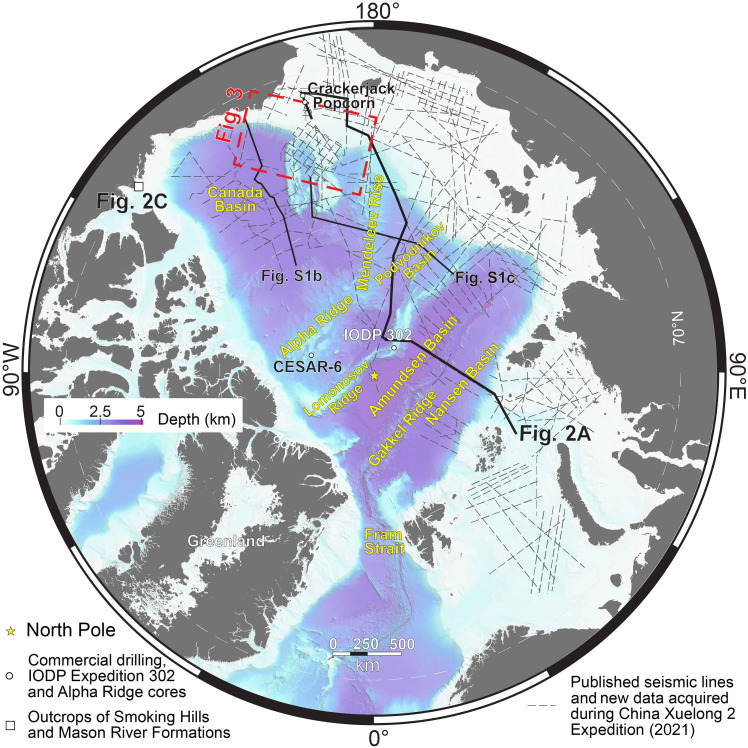
Bathymetry and data locations in the Arctic region. Bathymetric map of the Arctic region, modified from Springer Nature (*65*) under a CC BY 4.0 license (https://creativecommons.org/licenses/by/4.0/). The map shows the distribution of key datasets. Locations of multichannel reflection seismic profiles (*9*, *59*), commercial drilling sites (*13*, *62*), International Ocean Discovery Program (IODP) drilling sites (*8*), the Alpha Ridge CESAR-6 core (*3*), and Lower Cretaceous marine outcrops in northern Alaska (*43*, *44*) are shown.

In this study, we present previously unidentified sedimentological and geophysical evidence that supports a more dynamic paleoceanographic regime in the Late Cretaceous Arctic Ocean, based on a comprehensive suite of datasets (Fig. 1). The occurrence of Campanian-to-Maastrichtian [~80 to 66 million years ago (Ma)] contourite drifts and bathyal carbonate mounds reflects persistent bottom-current activity and high biological productivity. Together, these results reveal a vigorous and productive marine system influenced by seaway-driven water mass exchange and density-controlled circulation.

## RESULTS

### Contourite depositional systems and carbonate mounds

**Fig. 2.**
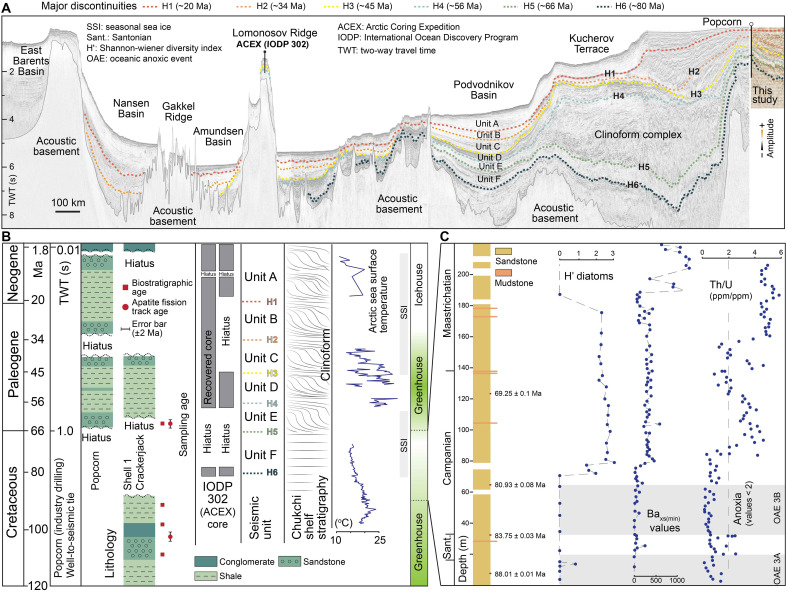
Seismic and chronostratigraphic framework and paleoproductivity of the Late Cretaceous Arctic. (**A**) Composite seismic profile across the Arctic Ocean, illustrating the stratigraphic framework. The depths and locations of main seismic horizons are based on the previous seismic framework (*10*). Location of the line is shown in Fig. 1. (**B**) Lithology and age constraints from the Popcorn and Crackerjack boreholes, reproduced from Geological Society of London (*13*). Hiatus depth in IODP Expedition 302 boreholes (*8*), major seismic units, Chukchi Shelf stratigraphy, and Arctic sea surface temperature records (*8*) are shown. (**C**) Paleoproductivity indicators (*43*, *44*), including diatom diversity, Ba_xs(min)_ values, and Th/U ratios from Campanian-to-Maastrichtian marine outcrops in northern Alaska. Outcrop location is indicated in Fig. 1. ppm, parts per million.

**Fig. 3.**
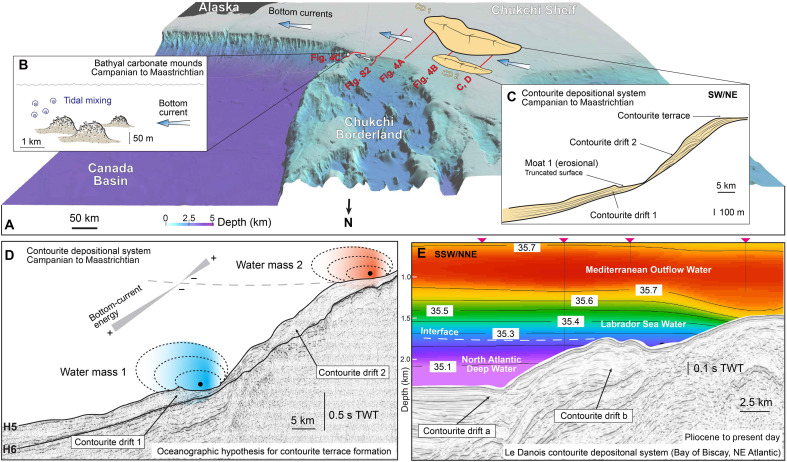
Campanian-to-Maastrichtian contourite drifts and bathyal carbonate mounds in the Arctic Ocean. (**A**) Location of selected seismic lines and spatial distribution of bottom-current–related features. (**B**) Dimensions and potential formation mechanisms of bathyal carbonate mounds. (**C** and **D**) Scale and seismic facies of contourite drifts 1 and 2, with a proposed formation hypothesis. (**E**) Oceanographic and seismic profiles of modern contourite drifts in the NE (northeast) Atlantic, shaped by two water masses. Colors (red to purple) represent seawater salinity variations, with salinity contour lines shown. Red triangle represents location of CTD (conductivity, temperature, and depth) stations. SSW, south-southwest; NNE, north-northeast.

The seismic stratigraphic framework is established through regional correlations with previous studies that identified key tectonostratigraphic transitions in the Arctic Ocean (Fig. 2) (*9*, *10*). Chronological constraints are derived from multiple data sources, including results from International Ocean Discovery Program (IODP) Expedition 302 on the Lomonosov Ridge, magnetostratigraphic data from the Eurasian Basin, and well logs from industry exploration wells (e.g., Popcorn and Crackerjack) located on the Chukchi continental shelf (Fig. 1; see Materials and Methods for details) (*10*–*13*). Key seismic discontinuities, H6 (~80 Ma), H5 (~66 Ma), H4 (~56 Ma), H3 (~45 Ma), H2 (~34 Ma), and H1 (~20 Ma), divide the stratigraphic record into six seismic units (units A to F). Bottom-current–related features described in this study occur within seismic unit F (~80 to 66 Ma) (Fig. 3), indicating a Campanian-to-Maastrichtian age.

**Fig. 4.**
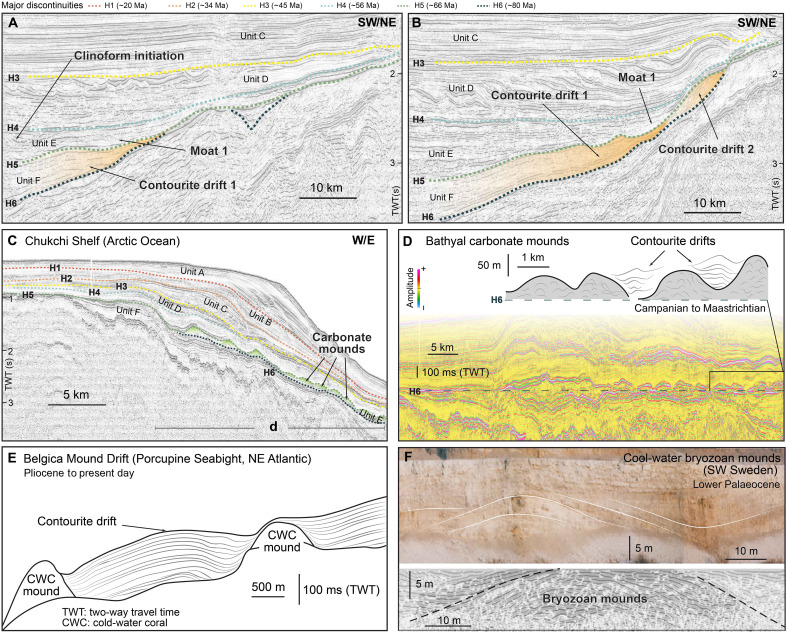
Seismic facies of contourite drifts and bathyal carbonate mounds. (**A** and **B**) Seismic interpretations of contourite drifts and (**C**) carbonate mounds. Locations of seismic lines are shown in Fig. 3. (**D**) Flattened seismic discontinuity H6 (~80 Ma) highlighting individual carbonate mounds. The profile location is indicated in Fig. 3C. (**E**) Interpretation of modern cold-water coral mounds and the Belgica Mound Drift in the Porcupine Seabight, shown as an analog. (**F**) Outcrop and seismic profiles of the cool-water bryozoan mounds in SW Sweden, reprinted with permission from (*25*) (copyright 2009 John Wiley and Sons).

Contourite drifts 1 and 2, notable sedimentary bodies formed by persistent bottom currents (*14*), are identified on the Chukchi Shelf, characterized by mounded geometries, moderate-amplitude seismic reflections, and sigmoidal to subparallel internal configurations (Fig. 4, A and B). These features trend northwest to southeast (Fig. 3). Contourite drift 1 is interpreted as a separated mounded drift of ~250 km in length, associated with an erosional moat (Moat 1) expressed by truncated seismic reflections. Contourite drift 2 is interpreted as a plastered drift accompanied by a contourite terrace (Figs. 3B and 4B), typically formed by the interaction of two distinct water masses, with the terrace marking the interface of these water masses (*15*). Comparable large-scale mounded contourite drifts and moats have not been identified at seismic resolution in other Arctic continental shelves or deep-sea regions (e.g., the Canadian Basin or Mendeleev Rise) within Late Cretaceous successions (Fig. 2A and fig. S1, B and C).

Several mounded features occur within Seismic Unit F (~80 to 66 Ma), rising 85 to 120 m above the paleo-seafloor and spanning 1.2 to 1.8 km in width (Fig. 4, C and D). These features exhibit steep flanks and are characterized internally by chaotic to semicontinuous seismic reflections, bounded by high-amplitude surfaces (Fig. 4, C and D). These mounds are interpreted as bathyal carbonate buildups (*16*) based on multiple lines of evidence. (i) Their seismic expression differs from alternative origins such as mud volcanoes, sediment waves, dunes, and mass-transport deposits, all of which display diagnostic geometries or internal structures absent here (*17*–*19*). (ii) Paleotemperature reconstructions indicate sea surface conditions of ~15° to 17°C (Fig. 2B) (*7*, *8*), compatible with cool-water carbonate accumulation (*20*) but well below the ~25° to 32°C required for tropical rudist–coral reef systems (*21*, *22*). (iii) The presence of small-scale contourite drifts associated with these mounds further indicates persistent bottom-current activity (Fig. 4D), providing favorable conditions for carbonate precipitation and mound accretion (*16*). (iv) The seismic characteristics of these mounds closely resemble cold-water coral mound provinces in other bathyal settings (Fig. 4E) (*23*) and the Cenomanian-to-Paleocene NW European epicontinental seas (Fig. 4F) (*24*, *25*), where these analogs typically exhibit similar dimensions and internal reflection patterns, as well as consistent associations with contourite drifts.

## DISCUSSION

### Multilayered oceanic circulation in the Late Cretaceous Arctic Ocean

The two distinct contourite drifts identified on the Chukchi Shelf reflect bottom-current processes and water-mass dynamics (Fig. 3D). Contourite drift 1 is associated with a pronounced erosional moat, whose formation requires bottom-current velocities exceeding ~16 cm/s based on flume tank experiments (*26*). Contourite drift 2 exhibits a contourite terrace morphology that typically forms at the interface between two water masses (*15*). Similar terrace-moat systems occur at the Le Danois Bank in the northeast Atlantic, where modern observations link their development to interactions between Labrador Sea Water and North Atlantic Deep Water (Fig. 3E) (*27*, *28*). By analogy, the two contourite drifts on the Chukchi Shelf imply the coexistence of at least two distinct water masses at different depths during the Campanian to Maastrichtian.

**Fig. 5.**
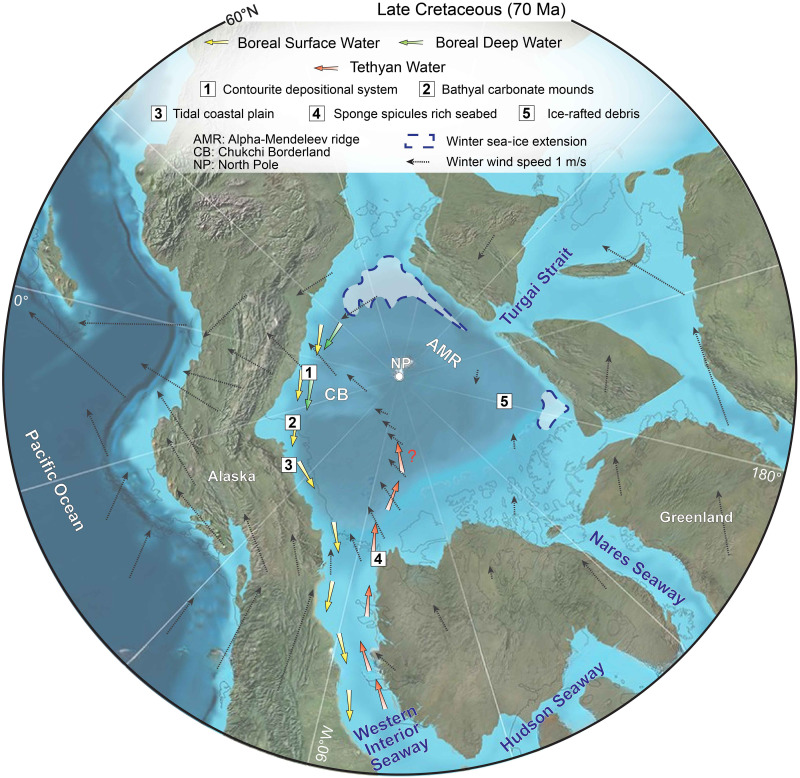
Late Cretaceous ocean circulation in the Arctic Ocean. The paleogeographic map at 70 Ma reproduced from the Atlantic Geoscience Society (*42*) under CC BY 4.0 license (https://creativecommons.org/licenses/by/4.0/). No modifications are made to the original image. Bottom current pathways are inferred from contourite features, while Tethyan Water pathways are reproduced with permission from Copernicus Publications (*29*), licensed under CC BY 4.0 (https://creativecommons.org/licenses/by/4.0/), and reprinted from Sedimentary Geology, volume 301, C. Schröder-Adams, The Cretaceous Polar and Western Interior seas: Paleoenvironmental history and paleoceanographic linkages, pages 26 to 40, copyright (2014), with permission from Elsevier (*30*). Winter winds and seasonal sea-ice extent are derived from simulation results (*36*).

Although oceanic circulation reconstructions for high-latitude regions of the Northern Hemisphere during this interval are rare, two shallow water masses have been documented: (i) cool, fresh Boreal Surface Water, influenced by substantial riverine input, and (ii) warm, saline Tethyan Water, sourced from the proto-Gulf of Mexico (*29*, *30*). Faunal assemblages and geochemical signatures from the Western Interior Seaway indicate that both water masses flowed at similar depths during the Campanian (fig. S2) (*30*). Moreover, Tethyan Water appears to have been largely confined to the Canadian margin (Fig. 5) (*5*). Paleogeographic reconstructions further suggest that the Chukchi Borderland may have been emergent during the Late Cretaceous (*31*), potentially acting as a topographic barrier to Tethyan inflow. Consequently, these shallow water masses are unlikely to generate the observed contourite system in the Arctic. An intermediate or deep-water mass is required to create the contourite terrace features observed on the Chukchi Shelf.

Recent studies have identified extensive contourite drifts, furrows, and erosional scours along the Greenland-Norwegian Seaway, suggesting active water-mass exchange between the North Atlantic and the Arctic Ocean during the Late Cretaceous (*32*, *33*). Calcareous nannofossil assemblages from northeast Greenland further suggest deposition in a deep-marine environment influenced by this water mass (*34*). However, the Lomonosov Ridge is thought to have been relatively shallow due to tectonic uplift during the Late Cretaceous (*31*). This paleogeographic configuration may have restricted the inflow of North Atlantic waters to the Chukchi Shelf. This implies the presence of an unrecognized deep-water mass below the Boreal surface water during this time.

Evidence of ice-rafted debris in the late Campanian section of core CESAR-6 from the Alpha Ridge (fig. S2) (*3*, *35*) together with climate model outputs (*36*) indicates episodic winter sea-ice formation west of the Alpha-Mendeleev ridge (Fig. 5). This interval is characterized by well-developed sediment laminations interpreted as repeated annual flux cycles associated with rafting and melting of turbid, sediment-laden sea ice (*3*, *35*, *37*). Although Late Cretaceous climate simulations suggest a warm, freshened Arctic surface ocean with substantial precipitation and runoff that largely precluded perennial sea ice (*2*), even short-lived seasonal freezing events along shelf margins can generate notable brine rejection and dense-water formation.

Modern observations from the Bering Sea, Chukchi Shelf, and Barents Sea demonstrate that short-lived sea-ice formation events in marginal ice zones can trigger dense-water cascades and initiate bottom-intensified currents (*38*–*40*). Observed current speeds associated with these cascades can reach ~15 cm/s (*39*), well within the range required to transport and redeposit fine-grained sediment to form contourite depositional systems (*26*). In the Late Cretaceous Arctic, restricted basin topography (*31*), strong tidal amplification across shelves (*41*), and winter wind–driven polynya activity (*36*) would have further enhanced vertical mixing and downslope dense-water export. A regionally sourced dense water mass, here interpreted as Boreal Deep Water, would have formed and deflected southward along the Eurasian continental margin by Coriolis forcing, sustaining vigorous bottom currents capable of sculpting the observed contourite drifts and mound complexes (Fig. 5).

Together, the evidence supports an interpretation in which contourite drift 1 records the activity of a regionally sourced Boreal Deep Water, whereas contourite drift 2 reflects interaction between fresh Boreal Surface Water and the sea-ice–driven Boreal deep water. Boreal deep water likely exerted only regional influence during this period. The enclosed nature of the Late Cretaceous Arctic deep basin (*42*) and the shallow Western Interior Seaway (*30*) would have restricted its penetration into lower latitudes. These findings indicate that the Late Cretaceous Arctic Ocean sustained a more complex, multilayered circulation system than previously recognized.

### Rise of bathyal carbonate mounds

Commercial drilling data, combined with paleontological analysis on the Chukchi Shelf (*13*), indicate that the location where bathyal carbonate mounds (~80 to 66 Ma) developed was a deep-shelf environment during the Campanian to Maastrichtian. Deep-shelf outcrops in northern Alaska reveal a notable increase in diatom diversity and elevated Ba content starting around 80 Ma (Fig. 2C) (*43*). Th/U ratios from the same period indicate a sharp increase in seawater oxygen levels on the Arctic shelf (Fig. 2C) (*44*). These changes suggest high paleoceanographic productivity and favorable nutrient conditions for bathyal carbonate mound accretion during the Campanian to Maastrichtian.

Beyond the Chukchi Shelf, seismic data across the Arctic reveal no comparable sedimentary features such as carbonate mounds (fig. S1), suggesting that their formation was driven by regionally specific processes. These likely include (i) seasonal sea-ice formation (*8*), which can enhance vertical nutrient fluxes and increase water column oxygenation (*45*); (ii) uplift of the Chukchi Borderland (*46*), where topographic highs could have created favorable conditions for localized upwelling (*39*), further promoting nutrient-rich water circulation; and (iii) intense tidal mixing in paleocoastal regions, such as northern Alaska (Fig. 5) (*41*), which may have further strengthened vertical and lateral seawater mixing. Together, these processes would have sustained high biological productivity and carbonate mound development on the Chukchi Shelf, while their absence elsewhere in the Arctic reflects the lack of such specific combination of environmental drivers.

The development of these bathyal carbonates occurred after Oceanic Anoxic Event 3 (OAE3) (Fig. 2C), marking a transition from widespread anoxic conditions to more oxygenated and dynamic oceanic systems (*44*). This temporal context suggests that the recovery of oxygenated conditions and enhanced circulation in the Arctic Ocean facilitated the establishment of bathyal carbonate ecosystems, analogous to present-day cold-water coral mound ecosystems. Growth of these carbonate mounds terminated in the latest Maastrichtian, coinciding with the Cretaceous-to-Paleogene mass extinction. Globally, carbonate mounds experienced a marked decline and ultimately vanished during this interval (*47*, *48*). The extinction of key planktonic and benthic taxa at the Cretaceous-to-Paleogene boundary disrupted carbonate and biogenic silica fluxes to the seafloor (*49*), altering sediment composition and influencing depositional patterns.

The Chukchi Shelf carbonate mounds thus represent a unique archive of Late Cretaceous Arctic palaeoceanography, recording localized productivity hot spots and oxygenation events in high-latitude marine settings. Their presence demonstrates that cool-water carbonate factories in a greenhouse Earth could flourish in polar shelf environments under specific combinations of nutrient availability, ocean circulation, and paleotopography. Moreover, these mounds provide a critical link between regional Arctic sedimentary dynamics and global carbonate deposition trends during the Late Cretaceous, highlighting the sensitivity of carbonate systems to both global oceanographic variations, such as OAE3 (*44*), and local paleoenvironmental factors.

### The “hiatus” problem

A pronounced stratigraphic hiatus is evident in most available Late Cretaceous–to–Paleogene Arctic Ocean drill sites. On the Chukchi Shelf, commercial drilling cores reveal missing intervals spanning ~120 to 80 to 66 to 60 Ma (*50*), while IODP Expedition 302 recovered a gap from ~80 to 56.2 Ma on the Lomonosov Ridge (*8*). In seismic reflection data, these hiatus top surfaces correspond to prominent erosional unconformities (discontinuities H5 and H4) traceable across much of the Arctic Ocean (Fig. 2A). Notably, major changes in sedimentary patterns, particularly in contourite features and the termination of bathyal carbonate development occurred around 66 to 65 Ma (Figs. 3 and 4). The widespread absence of sedimentary accumulation during this interval points to a prolonged phase of nondeposition or erosion, potentially linked to major global climatic and environmental changes.

The persistence of these hiatuses suggests a combination of tectonic, oceanographic, and climatic drivers. Because of Laramide tectonism and a eustatic sea level fall (*4*), the Western Interior Seaway closed during the latest Maastrichtian (~66 Ma) (*5*, *6*), fundamentally altering Arctic Ocean circulation patterns. As a result, the Arctic Ocean became more enclosed and cooled, as a region of net freshwater input (*2*). This gateway restriction reshaped basin connectivity and water mass exchange between the Arctic Ocean and the equatorial Atlantic-Tethys (*29*), thereby reducing heat transfer between low- and high-latitude regions.

These regional developments occurred within the context of major global reorganizations in thermohaline circulation identified in deep-sea records from outside the Arctic. Previous studies have shown that the Late Cretaceous–to–Paleogene transition was marked by marked shifts in deep-water sources and hiatus distribution in the world ocean (*51*). Isotopic proxy records (*52*) and sedimentary records (*53*) indicate that global deep-water pathways were reconfigured during this interval. A principal driver of these changes was the progressive opening and deepening of Atlantic gateways, which altered interbasin connectivity and facilitated more vigorous overturning circulation (*2*, *53*, *54*). Numerical simulations suggest that the initiation of a more globally integrated thermohaline circulation after the early Maastrichtian was closely tied to the opening of the South Atlantic and rearrangement of exchange between the Atlantic, Tethys, and Southern Ocean (*54*).

Viewed within this global framework, the Arctic hiatuses appear to be part of a broader reorganization of ocean circulation linked to both gateway tectonics and large-scale shifts in thermohaline circulation patterns. These gateway tectonics and paleoceanographic changes may have strengthened bottom currents (*55*), generating widespread current-scoured erosional surfaces, analogous to those associated with the Neogene closure of the South China Sea (*56*). Meanwhile, the latest Cretaceous transition from a warm greenhouse climate to a cooler icehouse, coupled with declining atmospheric temperatures (*57*), may have enhanced high-latitude deep-water formation, consistent with Late Cretaceous oxygen isotope evidence from the Southern Ocean (*58*). These processes likely invigorated Arctic ocean circulation, promoting sediment erosion and producing the widespread hiatus observed in the Arctic Ocean.

In summary, these processes produced a long-lived erosional regime in the Arctic Ocean, interrupting sediment accumulation over tens of millions of years and leaving a conspicuous imprint on both the seismic and stratigraphic record. The resulting unconformities not only serve as markers of Arctic Ocean paleoceanographic and tectonic evolution but also provide a framework to link regional sedimentary dynamics with global changes in sea level, climate, and biotic turnover. In this context, the Arctic hiatuses exemplify the complex interplay between global greenhouse-to-icehouse transitions, oceanic gateway tectonics, and ocean circulation reorganization, highlighting the Arctic Ocean as a sensitive recorder of Late Cretaceous–to–Paleogene environmental perturbations.

Nonetheless, limitations remain in our interpretations, particularly regarding the proposed mechanism of sea-ice–driven deep-water formation, which relies on indirect evidence from seismic features, ice-rafted debris proxies, and climate simulations (*3*, *35*, *36*). While physically plausible based on modern analogs, such as seasonal brine rejection (*38*–*40*), the extent to which intermittent sea ice could sustain bottom currents sufficient to form contourite drifts requires further validation through high-resolution ocean-atmosphere models. Integration of additional proxy data, such as neodymium isotopes from Arctic cores, could provide stronger constraints on water-mass sourcing and circulation patterns, building on global reconstructions. To apply these findings more broadly, future steps should include expanded seismic surveys across underexplored Arctic margins, targeted drilling to recover Campanian-to-Maastrichtian sediments for direct paleontological and geochemical analysis, and coupled simulations to test the sensitivity of circulation patterns to tectonic and climatic forcings.

## MATERIALS AND METHODS

### Datasets

Seismic data were acquired during the Russian Arktika expeditions in 2011, 2012, and 2014, aboard the R/V Akademik Fedorov (*9*); the US-led MGL1112 expedition MGL1112 in 2011, aboard the R/V Marcus G. Langseth (*11*); the Chinese 12th Arctic expedition in 2021 aboard the R/V Xuelong 2 (*59*); and various other seismic surveys conducted by the United States Geological Survey and the Geological Survey of Canada (*60*, *61*). Multiple reflections were predicted and removed primarily using the wave-equation multiple attenuation technique. Low-velocity surface waves, impulse, and irregular noise were attenuated. Seismic stacking deconvolution, spectral amplitude, balancing, and two-way coherency filtering were applied. All seismic datasets were processed to a high standard, ensuring their suitability for interpretation.

Commercial drilling wells Popcorn and Crackerjack, operated by Shell between 1989 and 1991, have been previously analyzed for lithology, age models, and seismic-to-well ties (*13*, *50*, *62*). Lithological and paleoceanographic proxy data were also obtained from IODP Expedition 302 (*8*), the Alpha Ridge CESAR-6 core (*3*, *35*, *37*), and Lower Cretaceous marine outcrops in the northern Alaska (*43*, *44*). The locations of all sedimentary records are shown in Fig. 1.

### Stratigraphic framework and chronology

The stratigraphic framework is established by correlation with previous studies (*9*–*13*). Seismic discontinuity H6 corresponds to the termination of volcanic activity on the Mendeleev Ridge, dated to ~80 Ma. Seismic discontinuity H5, marking the Cretaceous-to-Paleocene boundary at ~66 Ma, corresponds to the base of the clinoform complex on the Chukchi Shelf (fig. S3) and the Middle Brookian unconformity identified in commercial drilling. Seismic discontinuity H4, dated to ~56 Ma, represents a breakup unconformity associated with the opening of the Amundsen and Nansen basins. IODP drilling and borehole data indicate that seismic discontinuity H3 is defined at ~45 Ma. Seismic discontinuities H2 (~34 Ma) and H1 (~20 Ma) are constrained by regional tectonic interpretations and seismic-to-well ties from commercial drilling.

### Seismic interpretation

Seismic reflection profiles are interpreted using IHS Kingdom Suite, with vertical scale expressed in two-way travel time (TWT). Contourite drifts were interpreted on the basis of the varying degrees of mounded geometries, continuous wavy or divergent to subparallel reflection configurations, and onlap reflection terminations (Figs. 3 and 4) (*14*).

Carbonate mounds are characterized by chaotic to semicontinuous reflections, steep flanks, and localized mound-like geometries with limited lateral extent and irregular internal reflection pattern (Figs. 3 and 4) (*16*, *22*, *63*). The time-to-depth conversion is expressed used to determine the height of individual carbonate mounds is based on a model derived from the “Arctic-2012” seismic reflection experiment (fig. S4), conducted from the Mendeleev Rise to the Chukchi Borderland (*64*). The model comprised two primary layers: (i) an upper sedimentary section extending from the surface to a 2.2-km depth, assigned a constant interval velocity of 1500 m/s (*v1*), and (ii) a deeper section from 2.2 to 3.2 km, assigned a constant interval velocity of 1900 m/s (*v2*). Time-to-depth conversion is expressed as

$$D=t\frac{v}{2}$$

where *D* (in meters) is the depth, *t* is the TWT (in seconds), and *v* is the seismic velocity (in meters per second).

## References

[1] W. W. Hay, Evolving ideas about the Cretaceous climate and ocean circulation. Cretac. Res. 29, 725–753 (2008).

[2] J. B. Ladant, C. J. Poulsen, F. Fluteau, C. R. Tabor, K. G. MacLeod, E. E. Martin, S. J. Haynes, M. A. Rostami, Paleogeographic controls on the evolution of Late Cretaceous ocean circulation. Clim. Past 16, 973–1006 (2020).

[3] A. Davies, A. E. S. Kemp, J. Pike, Late Cretaceous seasonal ocean variability from the Arctic. Nature 460, 254–258 (2009). 19587768 10.1038/nature08141

[4] C. R. Scotese, C. Vérard, L. Burgener, R. P. Elling, A. T. Kocsis, The Cretaceous world: Plate tectonics, palaeogeography and palaeoclimate. Geol. Soc. London Spec. Publ. 544, 31–202 (2025).

[5] C. Schröder-Adams, The Cretaceous Polar and Western Interior seas: Paleoenvironmental history and paleoceanographic linkages. Sediment. Geol. 301, 26–40 (2014).

[6] C. Gaina, M. Jakobsson, E. O. Straume, M.-L. Timmermans, K. Boggild, S. Bünz, V. Schlindwein, A. Døssing, Arctic Ocean bathymetry and its connections to tectonics, oceanography and climate. Nat. Rev. Earth Environ. 6, 211–227 (2025).

[7] H. C. Jenkyns, A. Forster, S. Schouten, J. S. Sinninghe Damsté, High temperatures in the Late Cretaceous Arctic Ocean. Nature 432, 888–892 (2004). 15602558 10.1038/nature03143

[8] R. Stein, The Late Mesozoic-Cenozoic Arctic Ocean climate and sea ice history: A challenge for past and future scientific ocean drilling. Paleoceanogr. Paleoclimatol. 34, 1851–1894 (2019).

[9] A. M. Nikishin, E. I. Petrov, S. Cloetingh, A. V. Korniychuk, A. F. Morozov, O. V. Petrov, V. A. Poselov, A. V. Beziazykov, S. G. Skolotnev, N. A. Malyshev, V. E. Verzhbitsky, H. W. Posamentier, S. I. Freiman, E. A. Rodina, K. F. Startseva, N. N. Zhukov, Arctic Ocean Mega Project: Paper 1 - Data collection. Earth Sci. Rev. 217, 103559 (2021).

[10] A. M. Nikishin, E. I. Petrov, S. Cloetingh, N. A. Malyshev, A. F. Morozov, H. W. Posamentier, V. E. Verzhbitsky, S. I. Freiman, E. A. Rodina, K. F. Startseva, N. N. Zhukov, Arctic ocean mega project: Paper 2 – Arctic stratigraphy and regional tectonic structure. Earth Sci. Rev. 217, 103581 (2021).

[11] I. Ilhan, B. J. Coakley, Meso–Cenozoic evolution of the southwestern Chukchi Borderland, Arctic Ocean. Mar. Petrol. Geol. 95, 100–109 (2018).

[12] V. A. Poselov, V. V. Butsenko, A. A. Kireev, O. E. Smirnov, S. M. Zholondz, “Seismic Stratigraphy of Sedimentary Cover,” in *Geologic Structures of the Arctic Basin*, A. Piskarev, V. Poselov, V. Kaminsky, Eds. (Springer, 2019), pp. 71–104.

[13] S. S. Drachev, D. W. Houseknecht, The North Chukchi – Podvodnikov and the Zhokhov – Wrangel composite tectono-sedimentary elements, East Siberian Arctic. Geol. Soc. London Mem. 57, 656–668 (2025).

[14] M. Rebesco, F. J. Hernández-Molina, D. Van Rooij, A. Wåhlin, Contourites and associated sediments controlled by deep-water circulation processes: State-of-the-art and future considerations. Mar. Geol. 352, 111–154 (2014).

[15] F. J. Hernández-Molina, S. Campbell, G. Badalini, P. Thompson, R. Walker, M. Soto, B. Conti, B. Preu, A. Thieblemont, L. Hyslop, E. Miramontes, E. Morales, Large bedforms on contourite terraces: Sedimentary and conceptual implications. Geology 46, 27–30 (2017).

[16] D. Hebbeln, E. Samankassou, Where did ancient carbonate mounds grow — In bathyal depths or in shallow shelf waters? Earth Sci. Rev. 145, 56–65 (2015).

[17] N. Wu, C. A. L. Jackson, H. D. Johnson, D. M. Hodgson, H. D. Nugraha, Mass-transport complexes (MTCs) document subsidence patterns in a northern Gulf of Mexico salt minibasin. Basin Res. 32, 1300–1327 (2020).

[18] T. Vandorpe, I. Martins, J. Vitorino, D. Hebbeln, M. García, D. Van Rooij, Bottom currents and their influence on the sedimentation pattern in the El Arraiche mud volcano province, southern Gulf of Cadiz. Mar. Geol. 378, 114–126 (2016).

[19] S. Delivet, B. Van Eetvelt, X. Monteys, M. Ribó, D. Van Rooij, Seismic geomorphological reconstructions of Plio-Pleistocene bottom current variability at Goban Spur. Mar. Geol. 378, 261–275 (2016).

[20] N. Thibault, R. Harlou, N. H. Schovsbo, L. Stemmerik, F. Surlyk, Late Cretaceous (late Campanian–Maastrichtian) sea-surface temperature record of the Boreal Chalk Sea. Clim. Past 12, 429–438 (2016).

[21] T. Steuber, M. Rauch, J.-P. Masse, J. Graaf, M. Malkoč, Low-latitude seasonality of Cretaceous temperatures in warm and cold episodes. Nature 437, 1341–1344 (2005). 16251961 10.1038/nature04096

[22] D. U. Schmid, R. R. Leinfelder, M. Nose, Growth dynamics and ecology of Upper Jurassic mounds, with comparisons to Mid-Palaeozoic mounds. Sediment. Geol. 145, 343–376 (2001).

[23] A. O. Matossian, D. Van Rooij, Morphosedimentary evolution of the Belgica Mound Drift: Controls on contourite depositional system development in association with cold-water coral mounds. Mar. Geol. 477, 107410 (2024).

[24] F. Surlyk “A cool water carbonate ramp with bryozoan mounds: Late Cretaceous-Danian of the danish basin”, in *Cool-Water Carbonates* (SEPM, Society for Sedimentary Geology, 1997), pp. 293–308.

[25] L. Nielsen, A. S. Von Brockdorff, M. Bjerager, F. Surlyk, Three-dimensional architecture and development of Danian bryozoan mounds at Limhamn, south-west Sweden, using ground-penetrating radar. Sedimentology 56, 695–708 (2009).

[26] H. Wilckens, J. T. Eggenhuisen, P. H. Adema, F. J. Hernández-Molina, R. S. Jacinto, E. Miramontes, Secondary flow in contour currents controls the formation of moat-drift contourite systems. Commun. Earth Environ. 4, 316 (2023).

[27] S. Liu, F. J. Hernández-Molina, S. Rodrigues, D. Van Rooij, Deep-water circulation in the northeast Atlantic during the mid- and Late Cretaceous. Geology 51, 515–520 (2023).

[28] S. Liu, D. Van Rooij, T. Vandorpe, C. González-Pola, G. Ercilla, F. J. Hernández-Molina, Morphological features and associated bottom-current dynamics in the Le Danois Bank region (southern Bay of Biscay, NE Atlantic): A model in a topographically constrained small basin. Deep-Sea Res. I Oceanogr. Res. Pap. 149, 103054 (2019).

[29] J. S. Eldrett, P. Dodsworth, S. C. Bergman, M. Wright, D. Minisini, Water-mass evolution in the Cretaceous Western Interior Seaway of North America and equatorial Atlantic. Clim. Past 13, 855–878 (2017).

[30] C. M. Lowery, R. M. Leckie, R. Bryant, K. Elderbak, A. Parker, D. E. Polyak, M. Schmidt, O. Snoeyenbos-West, E. Sterzinar, The Late Cretaceous Western Interior Seaway as a model for oxygenation change in epicontinental restricted basins. Earth Sci. Rev. 177, 545–564 (2018).

[31] T. O. Sømme, A. G. Doré, E. R. Lundin, B. O. Tørudbakken, Triassic–Paleogene paleogeography of the Arctic: Implications for sediment routing and basin fill. AAPG Bull. 102, 2481–2517 (2018).

[32] F. M. Gradstein, M. A. Kaminski, F. P. Agterberg, Biostratigraphy and paleoceanography of the Cretaceous seaway between Norway and Greenland. Earth-Sci. Rev. 46, 27–98 (1999).

[33] C. Serié, I. Polonio, S. Thomas, L. Rojo, paper presented at the International Association of Sedimentologists Meeting 2024, Aberdeen, UK, 25–27 June 2024.

[34] S. Pauly, J. Mutterlose, P. Alsen, Early Cretaceous palaeoceanography of the Greenland–Norwegian Seaway evidenced by calcareous nannofossils. Mar. Micropaleontol. 90-91, 72–85 (2012).

[35] A. Davies, A. E. S. Kemp, Late Cretaceous seasonal palaeoclimatology and diatom palaeoecology from laminated sediments. Cretac. Res. 65, 82–111 (2016).

[36] I. Niezgodzki, J. Tyszka, G. Knorr, G. Lohmann, Was the Arctic Ocean ice free during the latest Cretaceous? The role of CO_2_ and gateway configurations. Global Planet. Change 177, 201–212 (2019).

[37] P. Mudie, S. Blasco, Lithostratigraphy of the CESAR cores. Geol. Surv. Can. Pap. 84-22, 59–99 (1985).

[38] V. V. Ivanov, G. I. Shapiro, Formation of a dense water cascade in the marginal ice zone in the Barents Sea. Deep Sea Res. Part 52, 1699–1717 (2005).

[39] R. S. Pickart, M. A. Spall, J. T. Mathis, Dynamics of upwelling in the Alaskan Beaufort Sea and associated shelf–basin fluxes. Deep Sea Res. Part I 76, 35–51 (2013).

[40] D. Nomura, H. Abe, T. Hirawake, A. Ooki, Y. Yamashita, A. Murayama, K. Ono, J. Nishioka, Formation of dense shelf water associated with sea ice freezing in the Gulf of Anadyr estimated with oxygen isotopic ratios. Prog. Oceanogr. 196, 102595 (2021).

[41] P. P. Flaig, P. J. McCarthy, A. R. Fiorillo, “A tidally influenced, high-latitude coastal-plain: The Upper Cretaceous (Maastrichtian) Prince Creek Formation, North Slope, Alaska,” in *From River to Rock Record: The preservation of fluvial sediments and their subsequent interpretation* (SEPM, Society for Sedimentary Geology, 2011), pp. 233–264.

[42] R. Blakey, Paleotectonic and paleogeographic history of the Arctic region. Atl. Geol. 57, 7–39 (2021).

[43] J. F. Diaz, L. Schwark, P. K. Pedersen, J. M. Galloway, M. Bringué, S. E. Grasby, Late Cretaceous ecosystem dynamics in the southern incipient Arctic Ocean: A micropaleontological and geochemical perspective. Global Planet. Change 244, 104643 (2025).

[44] S. E. Grasby, J. L. Crowley, M. T. Mohr, J. B. Percival, O. H. Ardakani, J. Galloway, M. Bringué, I. R. Smith, W. Yuan, Oceanic anoxic event 3 in Arctic Canada—Arc volcanism and ocean fertilization drove anoxia. GSA Bull. 137, 411–426 (2024).

[45] K. R. Arrigo, D. K. Perovich, R. S. Pickart, Z. W. Brown, G. L. van Dijken, K. E. Lowry, M. M. Mills, M. A. Palmer, W. M. Balch, F. Bahr, N. R. Bates, C. Benitez-Nelson, B. Bowler, E. Brownlee, J. K. Ehn, K. E. Frey, R. Garley, S. R. Laney, L. Lubelczyk, J. Mathis, A. Matsuoka, B. G. Mitchell, G. W. K. Moore, E. Ortega-Retuerta, S. Pal, C. M. Polashenski, R. A. Reynolds, B. Schieber, H. M. Sosik, M. Stephens, J. H. Swift, Massive phytoplankton blooms under arctic sea ice. Science 336, 1408–1408 (2012). 22678359 10.1126/science.1215065

[46] A. M. Nikishin, K. F. Aleshina, E. A. Rodina, G. R. Foulger, H. W. Posamentier, E. R. Chizhova, Tectonic evolution of the Amerasia Basin, Arctic Ocean. Gondwana Res. 146, 173–199 (2025).

[47] G. P. Eberli, D. Bernoulli, A. Vecsei, R. Sekti, M. Grasmueck, T. Lüdmann, F. S. Anselmetti, M. Mutti, G. D. Porta, A Cretaceous carbonate delta drift in the Montagna della Maiella, Italy. Sedimentology 66, 1266–1301 (2019).

[48] A. Pohl, Y. Donnadieu, Y. Godderis, C. Lanteaume, A. Hairabian, C. Frau, J. Michel, M. Laugie, J. J. G. Reijmer, C. R. Scotese, J. Borgomano, Carbonate platform production during the Cretaceous. GSA Bull. 132, 2606–2610 (2020).

[49] S. Esmeray-Senlet, J. D. Wright, R. K. Olsson, K. G. Miller, J. V. Browning, T. M. Quan, Evidence for reduced export productivity following the Cretaceous/Paleogene mass extinction. Paleoceanography 30, 718–738 (2015).

[50] W. H. Craddock, D. W. Houseknecht, Cretaceous–Cenozoic burial and exhumation history of the Chukchi Shelf, offshore Arctic Alaska. AAPG Bull. 100, 63–100 (2016).

[51] T. D. Frank, M. A. Arthur, Tectonic forcings of Maastrichtian ocean-climate evolution. Paleoceanography 14, 103–117 (1999).

[52] E. Pucéat, C. Lécuyer, L. Reisberg, Neodymium isotope evolution of NW Tethyan upper ocean waters throughout the Cretaceous. Earth Planet. Sci. Lett. 236, 705–720 (2005).

[53] F. J. Hernández-Molina, C. R. Scotese, Mesozoic and Cenozoic oceanic gateway evolution and global tectonics: Paleoceanographic and sedimentary implications. Geol. Soc. London Spec. Publ. 553, doi.org/10.1144/gslspecpub2025-1 (2026).

[54] Y. Donnadieu, E. Pucéat, M. Moiroud, F. Guillocheau, J.-F. Deconinck, A better-ventilated ocean triggered by Late Cretaceous changes in continental configuration. Nat. Commun. 7, 10316 (2016). 26777897 10.1038/ncomms10316 PMC4735640

[55] Z. L. Ng, S. Liu, H. Chen, S. Yin, F. J. Hernández-Molina, D. F. P. Duarte, X. Xue, Z. Lin, K. Luo, M. Su, Tectonic influence on the characteristics of contourite systems. Earth Sci. Rev. 272, 105327 (2026).

[56] S. Liu, H. Chen, M. Su, K. Luo, J. Wu, Y. Gao, Z. Meng, S. Rodrigues, D. Duarte, Z. L. Ng, Z. Sun, H. Zhuo, X. Xie, South China Sea records Late Miocene reorganization of western Pacific deep circulation. Nat. Commun. 15, 10228 (2024). 39587100 10.1038/s41467-024-54739-4 PMC11589845

[57] C. Linnert, S. A. Robinson, J. A. Lees, P. R. Bown, I. Pérez-Rodríguez, M. R. Petrizzo, F. Falzoni, K. Littler, J. A. Arz, E. E. Russell, Evidence for global cooling in the Late Cretaceous. Nat. Commun. 5, 4194 (2014). 24937202 10.1038/ncomms5194 PMC4082635

[58] D. P. Murphy, D. J. Thomas, The evolution of Late Cretaceous deep-ocean circulation in the Atlantic basins: Neodymium isotope evidence from South Atlantic drill sites for tectonic controls. Geochem. Geophys. Geosyst. 14, 5323–5340 (2013).

[59] R. Hutchinson Deborah, W. Houseknecht David, C. Mosher David, Canada Basin tectono-sedimentary element, Arctic Ocean. Geol. Soc. London Mem. 57, 1016–1028 (2025).

[60] D. C. Mosher, D. R. Hutchinson, “Canada Basin,” in *Geologic Structures of the Arctic Basin*, A. Piskarev, V. Poselov, V. Kaminsky, Eds. (Springer, 2019), pp. 295–325.

[61] T. Zhang, J. Li, X. Niu, W. Ding, Y. Fang, J. Lin, Y. Wang, C. Zha, P. Tan, F. Kong, J. Chen, X. Wei, J. Lu, J. Dyment, J. P. Morgan, Highly variable magmatic accretion at the ultraslow-spreading Gakkel Ridge. Nature 633, 109–113 (2024). 39169191 10.1038/s41586-024-07831-0 PMC11374676

[62] N. Kumar, J. W. Granath, P. A. Emmet, J. A. Helwig, M. G. Dinkelman, Chapter 33 Stratigraphic and tectonic framework of the US Chukchi Shelf: Exploration insights from a new regional deep-seismic reflection survey. Geol. Soc. London Mem. 35, 501–508 (2011).

[63] A. Freiwald, “Cold-water coral reefs,” in *Encyclopedia of Modern Coral Reefs*. (Springer, 2011), pp. 225–229.

[64] S. N. Kashubin, O. V. Petrov, I. M. Artemieva, A. F. Morozov, D. V. Vyatkina, Y. S. Golysheva, T. V. Kashubina, E. D. Milshtein, A. V. Rybalka, Y. M. Erinchek, T. S. Sakulina, N. A. Krupnova, A. A. Shulgin, Crustal structure of the Mendeleev Rise and the Chukchi Plateau (Arctic Ocean) along the Russian wide-angle and multichannel seismic reflection experiment “Arctic-2012”. J. Geodyn. 119, 107–122 (2018).

[65] M. Jakobsson, L. A. Mayer, C. Bringensparr, C. F. Castro, R. Mohammad, P. Johnson, T. Ketter, D. Accettella, D. Amblas, L. An, J. E. Arndt, M. Canals, J. L. Casamor, N. Chauché, B. Coakley, S. Danielson, M. Demarte, M.-L. Dickson, B. Dorschel, J. A. Dowdeswell, S. Dreutter, A. C. Fremand, D. Gallant, J. K. Hall, L. Hehemann, H. Hodnesdal, J. Hong, R. Ivaldi, E. Kane, I. Klaucke, D. W. Krawczyk, Y. Kristoffersen, B. R. Kuipers, R. Millan, G. Masetti, M. Morlighem, R. Noormets, M. M. Prescott, M. Rebesco, E. Rignot, I. Semiletov, A. J. Tate, P. Travaglini, I. Velicogna, P. Weatherall, W. Weinrebe, J. K. Willis, M. Wood, Y. Zarayskaya, T. Zhang, M. Zimmermann, K. B. Zinglersen, The International Bathymetric Chart of the Arctic Ocean Version 4.0. Sci. Data 7, 176 (2020). 32647176 10.1038/s41597-020-0520-9 PMC7347603

