# Supplementary material for: High productivity and multilayered circulation in the Late Cretaceous Arctic Ocean
Source: Sci Adv. 2026 Mar 6;12(10):eaec4895. doi: 10.1126/sciadv.aec4895 (PMC13267304; doi:10.1126/sciadv.aec4895)
Supplement: Supplementary file 1 — Supplementary Text Figs. S1 to S4 [file sciadv.aec4895_sm.pdf]

Science Advances
AAAS

Supplementary Materials for

High productivity and multilayered circulation in the Late Cretaceous Arctic Ocean

Shan Liu *et al.*

Corresponding author: Shan Liu, liush296@mail.sysu.edu.cn; Haiteng Zhuo, zhuoht3@mail.sysu.edu.cn

Sci. Adv. **12**, eaec4895 (2026)
DOI: 10.1126/sciadv.aec4895

This PDF file includes:

Supplementary Text
Figs. S1 to S4

Supplementary Text

Supplementary figures

Figures S1–S4 provide a detailed view of Arctic Ocean sedimentary and tectonic features, highlighting both regional and basin-scale processes. **Fig. S1** presents seismic units and interpretations across the Arctic, including contourite drifts on the Chukchi Shelf (a), the abyssal sedimentary succession of the Canadian Basin (b), and seismic units in the Provodnikov Basin and Mendeleev Rise (c), with line locations shown in Figures 1 and 3. **Fig. S2** illustrates ice-rafted from the CESAR-6 core on Alpha Ridge (a) and the Cretaceous tectonic evolution of the Western Interior Seaway, indicating water-mass distribution and oxygenation levels (b). **Fig. S3** shows the seismic stratigraphic framework of the Chukchi Shelf, highlighting shelf-edge trajectories and clinoform development. **Fig. S4** presents seismic velocity models from the "Arctic-2012" experiment, including both multi-channel and wide-angle data, providing constraints on subsurface structure and sediment properties.

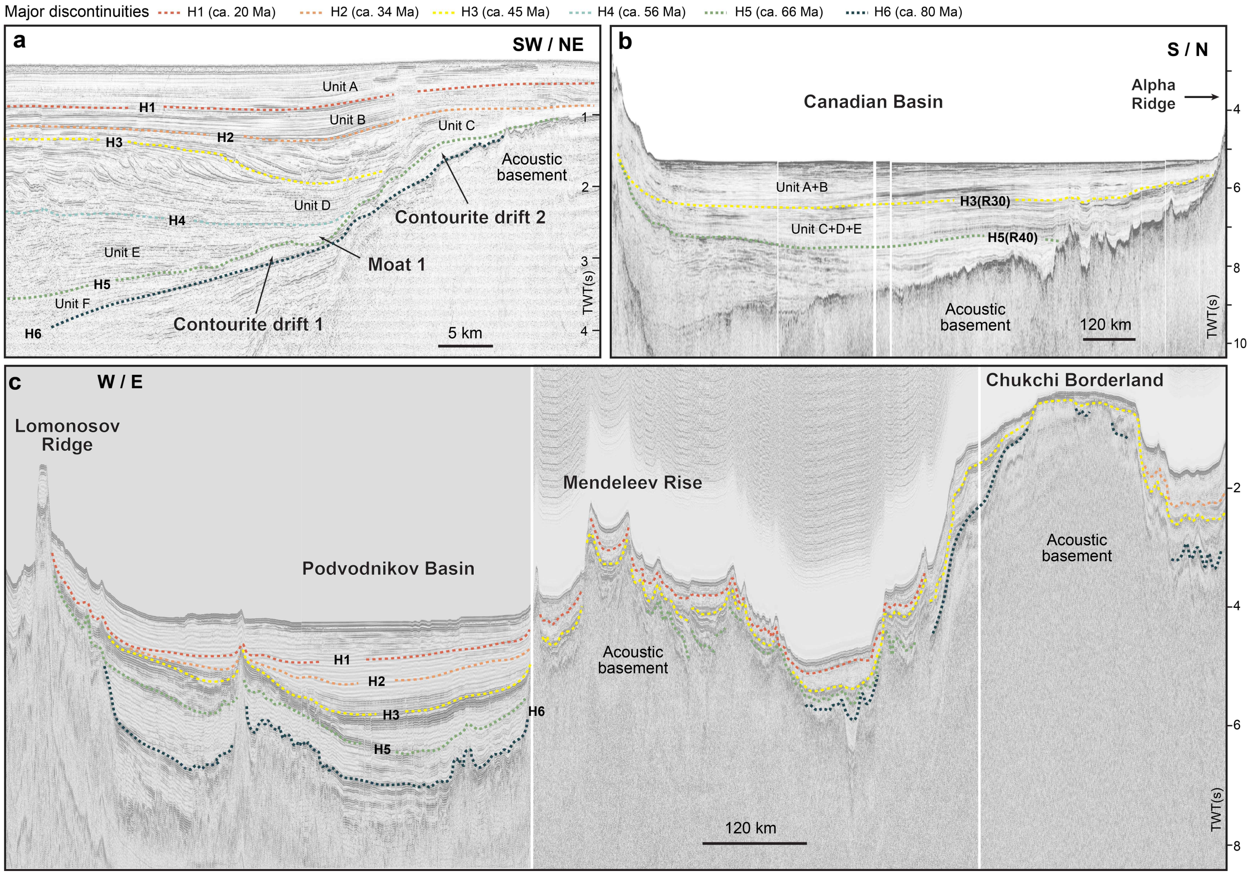

Fig. S1. Seismic units and interpretation in the Arctic Ocean. (A) Contourite drifts on the Chukchi Shelf. **(B)** Abyssal sedimentary succession in the Canadian Basin. **(C)** Seismic units in the Provodnikov Basin and on the Mendeleev Rise. Location of the seismic lines are shown in Figs. 1 and 3.

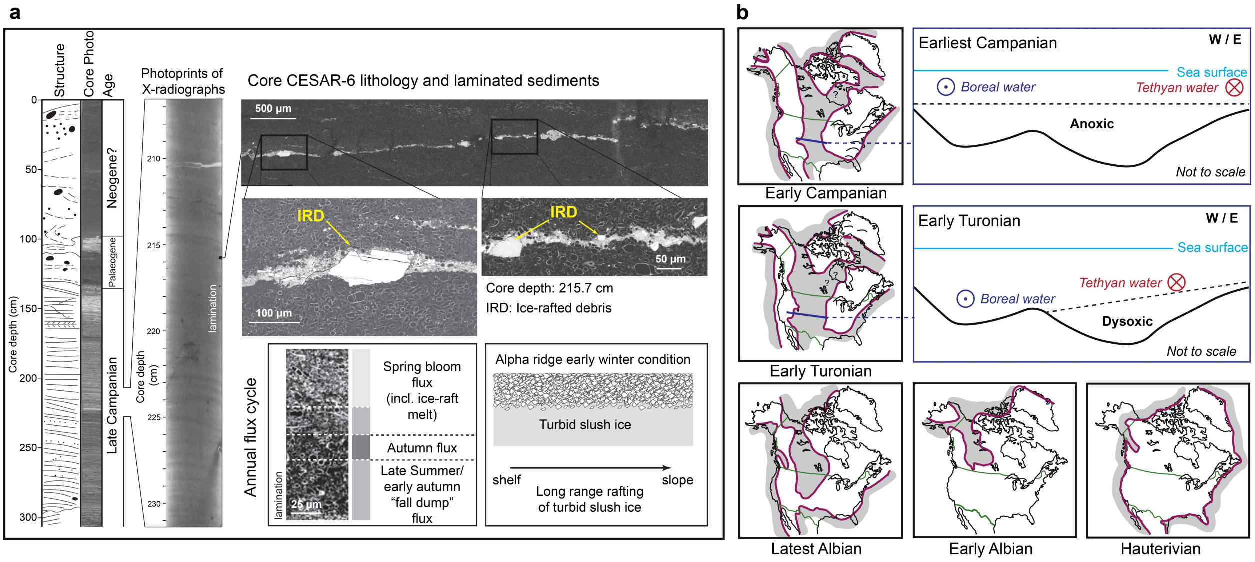

Fig. S2. Ice-rafted debris and tectonic evolution of the Western Interior Seaway. (A) Lithology, core image, and X-ray photoprints from CESAR-6 on the Alpha Ridge, reproduced with permission from Springer Nature (*3*) under CC BY 4.0 license and reprinted from Cretaceous Research, Vol. 65, Andrew Davies, Alan E.S. Kemp, Late Cretaceous seasonal palaeoclimatology and diatom palaeoecology from laminated sediments, Pages 82-111, Copyright (2016), with permission from Elsevier (*35*). The core shows laminated sediments that are most prominent in the Late Campanian interval, reflecting repeated annual flux cycles associated with seasonal sea-ice rafting and melt processes. **(B)** Cretaceous tectonic evolution of the Western Interior Seaway and water mass distribution. Reprinted from Earth-Science Reviews, Vol 177, Christopher M. Lowery et al., The Late Cretaceous Western Interior Seaway as a model for oxygenation change in epicontinental restricted basins, Pages 545-564, Copyright (2018), with permission from Elsevier (*5*); and reprinted from Sedimentary Geology, Vol 301, Claudia Schröder-Adams, The Cretaceous Polar and Western Interior seas: paleoenvironmental history and paleoceanographic linkages, Pages 26-40, Copyright (2014), with permission from Elsevier (*30*).

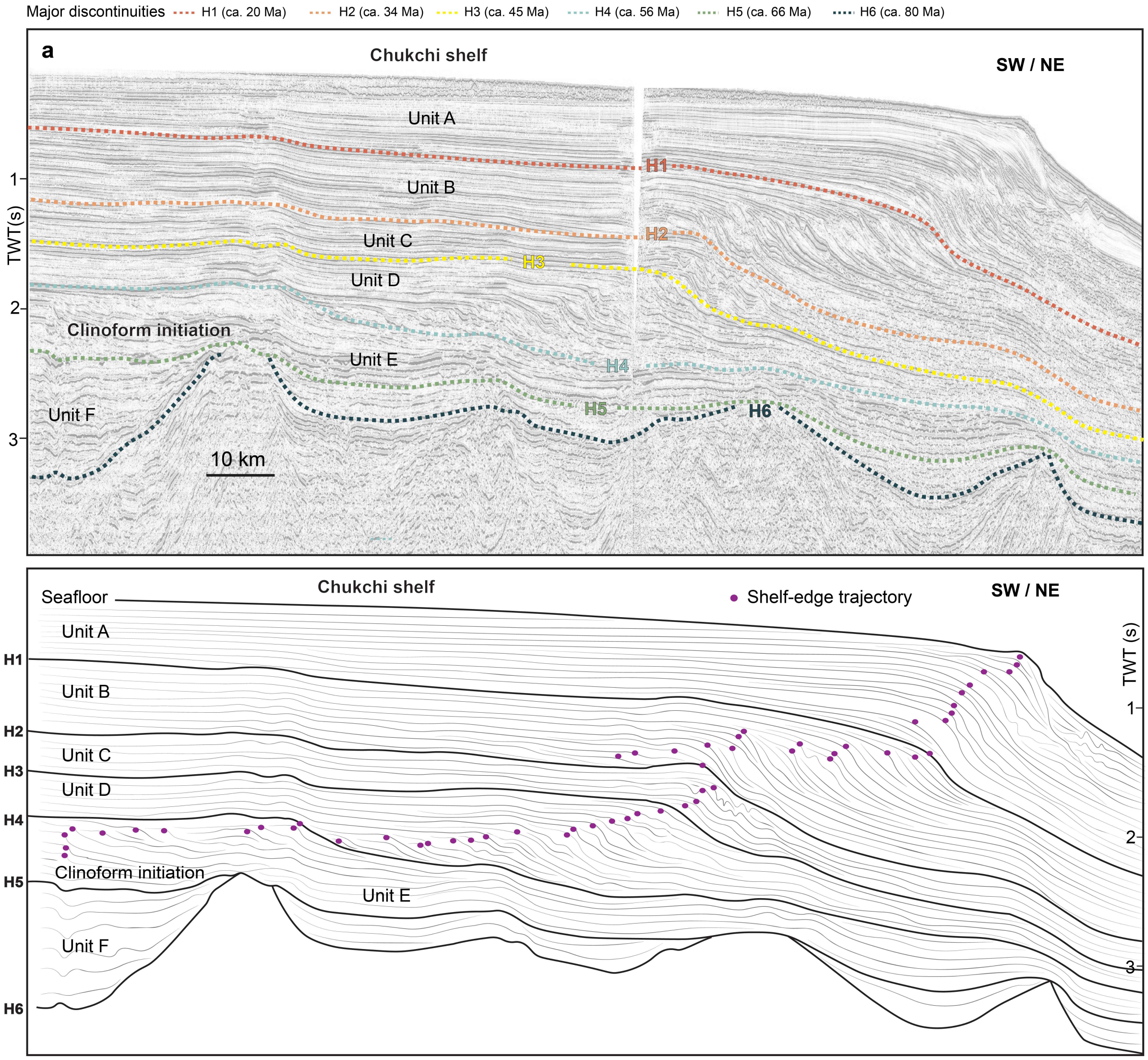

Fig. S3. Seismic stratigraphic framework of the Chukchi Shelf. Shelf-edge trajectory and clinoform development are indicated.

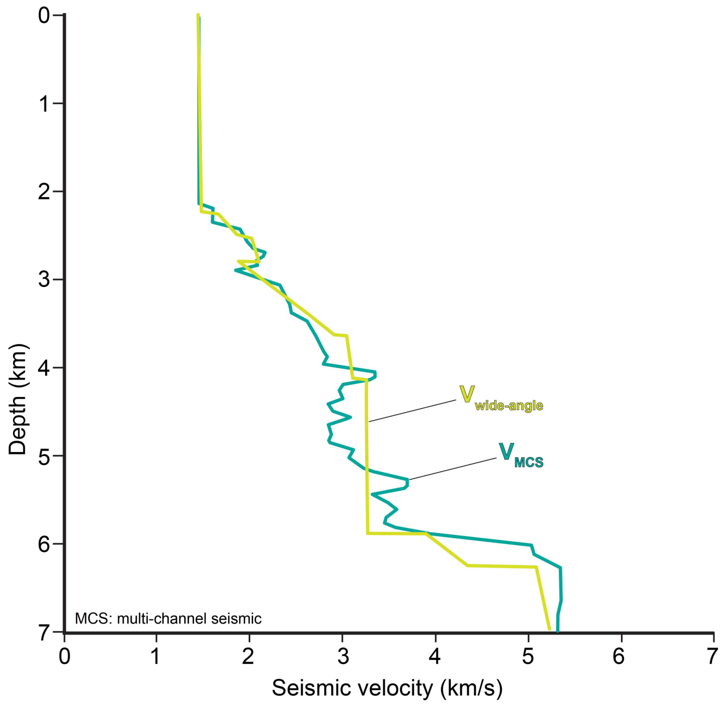

Fig. S4. Seismic velocity model from the "Arctic-2012" seismic reflection experiment. Velocity data (*64*) for multi-channel and wide-angle seismic are shown.
